# Prevalence of abdominal obesity in adolescents: association between sociodemographic factors and lifestyle

**DOI:** 10.1016/j.rppede.2016.01.007

**Published:** 2016

**Authors:** João Antônio Chula Castro, Heloyse Elaine Gimenes Nunes, Diego Augusto Santos Silva

**Affiliations:** a Programa de Pós-Graduação em Educação Física, Universidade Federal de Santa Catarina (UFSC), Florianópolis, SC, Brazil

**Keywords:** Waist circumference, Lifestyle, Anthropometry, Epidemiology, Adolescent health, Public health

## Abstract

**Objective:**

To estimate the prevalence of abdominal obesity and verify the association with sociodemographic factors (gender, school shift, ethnicity, age, maternal education and economic status) and lifestyle (alcohol consumption, sleep, soft drink consumption, level of physical activity and sedentary behavior) in adolescents in Southern Brazil.

**Methods:**

This was a cross-sectional epidemiological study of 930 adolescents (490 girls) aged 14–19 years, living in the city of São José, SC, Brazil. A self-administered questionnaire was used to collect sociodemographic and lifestyle data. Abdominal obesity was measured through the waist circumference and analyzed according to gender and age. Descriptive statistics (absolute and relative frequency, mean and standard deviation) and binary logistic regression, expressed as *Odds Ratios* (OR) and 95% confidence interval (95%CI) were employed, with *p*<0.05 being considered statistically significant; the SPSS 17.0 software was used for the statistical analyses.

**Results:**

The prevalence of abdominal obesity was 10.6% for the total sample (10.5% male, 10.8% female). Adolescents that watched television daily for two or more hours (OR=2.11, 95%CI 1.08–4.13) had a higher chance of having abdominal obesity and adolescents whose mothers had fewer than eight years of schooling (OR=0.56; 95%CI from 0.35 to 0.91) had a lower chance of having abdominal obesity.

**Conclusions:**

Approximately one in 10 adolescents had abdominal obesity; the associated factors were maternal schooling (≥8 years) and television screen time (≥2h/day).

## Resumo

**Objetivo:**

Estimar a prevalência de obesidade abdominal e verificar a associação com fatores sociodemográficos (sexo, turno de estudo, cor da pele, idade, escolaridade materna e nível econômico) e o estilo de vida (consumo de álcool, sono, consumo de refrigerante, nível de atividade física e comportamento sedentário) em adolescentes do Sul do Brasil.

**Métodos:**

Estudo epidemiológico descritivo transversal, feito com 930 adolescentes (490 do sexo feminino) de 14-19 anos de São José, SC, Brasil. Usou-se questionário autoadministrado para coletar dados sociodemográficos e do estilo de vida. A obesidade abdominal foi avaliada pelo perímetro da cintura e analisada de acordo com sexo e idade. Empregou-se estatística descritiva (frequência absoluta e relativa, média e desvio padrão) e regressão logística binária, expressa em *odds ratio* (OR) e intervalo de confiança de 95% (IC95%), foi significativo *p*<0,05 e usou-se o *software SPSS* 17.0.

**Resultados:**

A prevalência de obesidade abdominal foi de 10,6% para mostra total (10,5% masculino; 10,8% feminino). Adolescentes que assistiam à televisão diariamente por duas ou mais horas (OR=2,11; IC95% 1,08-4,13) apresentaram maiores chances de obesidade abdominal e os adolescentes cujas mães tinham escolaridade inferior a oito anos (OR=0,56; IC95% 0,35-0,91) tiveram menor chance de obesidade abdominal.

**Conclusões:**

Aproximadamente um a cada 10 adolescentes apresentou obesidade abdominal, os fatores associados foram a escolaridade materna (≥8 anos) e o tempo de tela de televisão (≥2 horas/dia).

## Introduction

Abdominal fat accumulation in adolescents is an independent risk factor for chronic disease, such as hypertension, fatty liver, insulin resistance, and type II diabetes,^1^^,^ 2 as well association with metabolic syndrome in adolescence and adulthood.^2^ Waist circumference (WC) is one way to assess abdominal obesity (AO), described as an anthropometric indicator of easy applicability and accuracy.^3^

The literature reports different prevalence of AO, which shows differences and/or cultural and social similarities.^4^ Park et al.^1^ found difference in prevalence of AO when comparing adolescents aged 12-19 years in the United States and South Korea (34.7% and 8.4%, respectively). Schröder et al.^5^ described AO prevalence of 11.6% when investigating Spanish adolescents aged 12-17 years. These differences in AO prevalence are also seen in Brazil. A study conducted with adolescents from Maranhão (Northeast region) showed a prevalence of 22.7%.^6^ Silva et al.^7^ in a study with 1065 adolescents (aged 14-17 years) found 2.1% of AO prevalence in the Southeast region (Minas Gerais) and 6.3% in the South region (Santa Catarina). Also in the South region, studies performed in Curitiba^8^ and Saudades^9^ found AO prevalence of 12.2% and 13.3% in adolescents, respectively.

Evidence of AO association with sociodemographic factors and lifestyle are still unclear. Although it is seen that female adolescents have higher percentages of body fat,^10^ there is a tendency in the literature to describe higher prevalence of AO in males,^5^^,^ 7 ^,^ 8 but there is no consensus on the relationship between AO and sex in adolescents.^4^ There are also discrepancies in the findings regarding the economic level, with studies showing a higher prevalence of AO in countries with higher economic levels,^4^ at the same time that investigations in regions with lower economic levels also showed a high prevalence of AO.^7^^,^ 8 Researches that found excessive consumption of soft drinks in adolescents found no association with AO,^11^^,^ 12 even knowing that inadequate diets and high sugar intake are associated with higher prevalence of AO.

Taking into account that AO entails risks to the health of adolescents and implications throughout life and that the possible combinations of AO with sociodemographic factors and lifestyle are not yet clear, the prevalence of AO in adolescents and possible associated factors should be investigated. The aim of this study was to estimate AO prevalence and its association with sociodemographic factors and lifestyle among adolescents in a city in the South region of Brazil.

## Method

The population of this epidemiological, cross-sectional study was composed of adolescents, aged 14-19 years, enrolled in high school in São José, Santa Catarina, Brazil. This study was approved by the Institutional Review Board of the Federal University of Santa Catarina (CAAE: 33210414.3.0000.0121).

The sample was determined in two stages: stratified by state public high schools (according to the number of students per school) and conglomerate classes, considering school shift and school grade. To determine the sample size, we followed the procedures suggested by Luiz and Magnanini,^13^ from the finite population. A population of 5182 students in 11 eligible schools and 170 classes distributed in high school grades (74.8% of the students attended day classes) was considered. We adopted a confidence level of 1.96 (95% confidence interval), tolerance error of 5%, 50% prevalence, and design effect of 1.5.^13^ We added 20% to minimize possible losses and refusals and another 20% to control for possible confounding variables in association studies.^14^ With these parameters, the required sample size was 751 students. Adolescents of both sexes, aged 14-19 years, attending public high schools of the City of São José, SC, Brazil, were eligible for the study. Pregnant adolescents, those who had children in the past six months, adolescents who did not give informed consent signed by parents (age <18 years) or by themselves (age ≥18 years), refused to participate, and those with physical disabilities that prevent the performance of the physical macroproject tests were not evaluated. After class conglomerate process in which all students in selected classes were collected and met the eligibility criteria, the total sample consisted of 1132 students.

Anthropometric measurements and understanding of the questionnaire were pretested, and a pilot study was conducted in July 2014 in Paulo Lopes, SC, Brazil, with 84 high school students who agreed to participate. Data collection in San Jose, SC, Brazil, was made from August to November 2014. To this end, seven post-graduate and four graduate students in physical education were selected, three of them had level 1 certification from the International Society for the Advancement of Kinanthropometry and made the anthropometric measurement.

WC was measured at the narrowest portion of the trunk, between the lower costal margin and the iliac crest, with anthropometric measuring tape.^3^ To classify adolescents with AO, the cutoff points proposed by Taylor et al.^3^ were used, which defined as excess fat values with *Z* score ≥1. These cutoffs points are near the 85th percentile, considered cut-off point for overweight by body mass index.^3^ Cutoffs points have been proposed according to age and sex (sensitivity and specificity values of 84% and 94% for female and 87% and 92% for male, respectively)^3^ (Table 1).

**Table 1:** Description of variables, instrument of measurement/question and categorization.

Type	Variables	Instrument/Question	Categorization	
Dependent	Waist circumference	Anthropometric tape	Cutoff points	
			Female:	Male:
			14 years≥77	14 years≥79
			15 years≥78.3	15 years≥81.1
			16 years≥79.1	16 years≥83.1
			17 years≥79.8	17 years≥84.9
			18 years≥80.1	18 years≥86.7
			19 years≥80.1	19 years≥88.4
			<Cutoff point=Normal	
			Cutoff points=Abdominal obesity^3^	

Independents	Sex	What is your sex?	Female/Male	
	Skin color	The Brazilian census uses the words white, brown, black, yellow and indigenous to classify the color or race of people. If you had to answer this question, how would you rate yourself about your color or ethnicity?	White	
			Black/Brown/Yellow/Indigenous^15^	
	Age	How old are you?	14-16 years	
			17-19 years[30]	
	Maternal education	What is the level of education completed by your mother?	<8 years	
			≥8 years^16^	
	Economic level	What is the monthly income of your family (the current minimum wage is R$724)?^17^	A/B=High	
			C/D/E=Low^9^	
	School shift	What is your school shift?	Morning/Afternoon/Full day=Day	
			Evening=Evening	
	Physical activity	During a typical week, on how many days you practice moderate to vigorous physical activity (physical activity during leisure time, at work, and going to school)?^18^	<5 days=Insufficiently active	
			≥5 days=Active^19^	
	Alcohol consumption	During the last 30 days, on how many days did you drink five or more alcoholic beverages in a single occasion?^18^	No day=No	
			≥1 day=Yes^29^	
	Soft drink consumption	During the last 7 days, how many times did you drink a bottle, can or glass of soda such as Coca-Cola, Fanta, Sprite, Pepsi and Pureza? (Do not consider diet and light soft drinks)^18^	I did not drink soft drinks in the last 7 days=No	
			Other options=Yes^12^	
	Sleep	Do you sleep well and feel rested?^20^	Almost never/Rarely/Sometimes=No	
			With relative frequency/Almost always=Yes^20^	
	TV Time	How many hours per day do you watch TV on school days (Monday to Friday)?	<2h/≥2h^19^	
		How many hours per day do you watch TV on weekends (Saturday and Sunday)?		
	Computer time	How many hours per day do you use the computer on school days (Monday to Friday)?	<2h/≥2h^19^	
		How many hours per day do you use computer on weekends (Saturday and Sunday)?		
	Video game time	How many hours per day you play video games on school days (Monday to Friday)?	<2h/≥2h^19^	
		How many hours per day you play video games on weekends (Saturday and Sunday)?		
	Total screen time	Total	<4hours/≥4hours^21^^,^ 22	
	Travel to school	How do you usually travel to school?	Walking/biking=Active	
			Car or motorcycle/bus=Passive^22^	

A self-administered questionnaire was applied with questions related to sociodemographic variables (gender, skin color, age, maternal education, economic level, and school shift) and lifestyle (physical activity, alcohol consumption, soft drink consumption, sleep, and sedentary behavior) (Table 1).

Categories of skin color were collected in accordance with the recommendations of the Brazilian Institute of Geography and Statistics^15^ and categorized in brown, black, yellow or red as a category, and white in another category.^15^ Maternal education was categorized taking into account the average schooling of Brazilians (7.2 years),^16^ classified as low (<8 years) and high (≥8 years). Economic level was evaluated by household purchasing power. For this study the categories A and B were defined as high and the remaining as low.^17^

To evaluate the consumption of alcohol and soft drinks and the level of physical activity (PA), questions of the Youth Risk Behavior Survey (YRBS) were used, translated and validated for Brazil.^18^ PA categorization took into account the evidence showing that 60min of PA five days per week is sufficient to maintain health in adolescence and higher amounts would provide additional benefits.^19^ For alcohol consumption, subjects were categorized into "no" (those who did not consume in the last 30 days five or more alcoholic drinks in one occasion) and "yes" (those who consumed such dosage in one or more days in the last 30 days). For soft drink consumption, adolescents were classified as "no" (those who did not consume any time during the week) and "yes" (those who consumed one or more times during the week).

Sleep was evaluated for quality using the Fantastic Lifestyle questionnaire, translated and validated for Brazil.^20^ Individuals who said they slept well "almost always" and "fairly often" were considered as good quality sleep (yes), while those who answered "almost never", "rarely", and "sometimes" were considered as poor quality sleep (no).

To assess sedentary behavior (SB) on weekdays and weekends, the 2-h cutoff point was used for each behavior, due to evidence of harm to the health of young people who have SB above that value.^19^ When behaviors are added, the multiplication of the 2-h cutoff point by 2 is used (totaling 4h) for total display time.^21^^,^ 22

Travel to school was classified as active for those with energy expenditure in this traveling or passive for those who traveled by car without energy expenditure.^22^

Descriptive statistics was used with analysis of absolute and relative frequency, mean and standard deviation. For comparison of medians, the Mann Whitney *U* test was used to compare frequencies, the chi-square test of heterogeneity. To identify factors associated with the dependent variable, binary logistic regression was used to estimate *Odds Ratio* and 95% confidence intervals. For all statistical tests, significance level was set at *p*<0.05. In regression analysis we used the hierarchical model of determination, hypothetically temporal, from distal to proximal determinants,^23^ in which the demographic variables (gender, skin color, and age) were included in the distal block, socioeconomic variables (economic level, maternal education, and school shift) in the intermediate block, and lifestyle variables (PA, alcohol consumption, soft drink consumption, sleep, and SB) in the distal block. In the regression model, all covariates were treated dichotomously, as described in Table 1 . The selection for input variables in the adjusted model was made using the backward method. All variables were included in the adjusted analysis, regardless of the crude analysis *p*-value. Adjustments were made for variables at the same level and above levels that represented *p*≤0.20 in the Wald test (adjusted analysis) and remained in the model.^24^ The SPSS 17.0 software was used for all analyzes.

## Results

There was a sample loss of 17.8% of adolescents, which included those who answered the questionnaire, but did not participate in the macroproject anthropometric assessment. Thus, the present study sample included 930 adolescents, mean age of 16.1±1.1 years, with female prevalence (*n*=490) (Table 2). Most adolescents had white skin color (62.7%), 14-16 years of age (58%), low maternal education (56.4%), high economic level (68.2%), and attended day school (69.8%) (Table 2). Nine out of 10 adolescents were little physically active (92.1%), three out of 10 consumed excessive alcohol (33.9%), eight out of 10 consumed soft drinks (84.1%), and about two thirds did not sleep well (61.4%). Regarding SB, seven out of 10 adolescents watched two or more hours of television (78.6%), about two-thirds of the students spent two or more hours on the computer (68.7%), and three out of 10 played video games for more than two hours (28%). Approximately nine out of 10 adolescents had screen time above 4h per day (87.2%) and half of the students used passive transportation to get to school (50.1%) (Table 3). Adolescents who were male, older, attended evening classes, drank alcohol and played video games daily for 2h or more had higher WC (*p*<0.05). There was no difference in AO prevalence between categories of the independent variables (Tables 2 and 3).

**Table 2:** Sample distribution, waist circumference (mean and standard deviation) and abdominal obesity prevalence according to sociodemographic variables.

Variables	Sample	Waist circumference	*p*-value	Abdominal obesity	*p*-value
	*n* (%)	M±SD		*n* (%)	
Total	930	71.5±8.0		99 (10.6)	
*Sex*					
Male	440 (47.3)	73.8±7.7		46 (10.5)	
Female	490 (52.7)	69.4±7.6	<0.01^a^	53 (10.8)	0.85

*Skin color*					
White	574 (62.7)	71.0±7.7		56 (9.8)	
Black/brown/yellow/red	342 (37.3)	72.0±8.3	0.09	41 (12.0)	0.28

*Age (years)*					
14-16	539 (58.0)	70.6±7.7		54 (10.0)	
17-19	391 (42.0)	72.7±8.2	<0.01^a^	45 (11.5)	0.46

*Maternal education*					
<8 years	518 (56.4)	71.3±8.0		49 (9.5)	
≥8 years	400 (43.6)	71.7±8.0	0.38	50 (12.5)	0.14

*Economic level*					
High	537 (68.2)	71.5±7.7		51 (9.5)	
Low	250 (31.8)	71.2±8.6	0.32	31 (12.4)	0.21

*School shift*					
Day	644 (69.8)	70.8±7.9		66 (10.2)	
Evening	278 (30.2)	72.9±8.0	<0.01^a^	33 (11.9)	0.46

M, mean; SD, standard deviation.

a *p*≤0.05; Mann-Whitney *U* test for independent samples.

**Table 3:** Sample distribution, waist circumference (mean and standard deviation) and abdominal obesity prevalence according to behavioral variables.

Variables	Sample	Waist circumference	*p*-value	Abdominal obesity	*p*-value
	*n* (%)	M±SD		*n* (%)	
Total	930	71.5±8.0		99 (10.6)	
*Physical activity*					
Insufficiently active	832 (92.1)	71.5±8.1		94 (11.3)	
Active	71 (7.9)	72.0±6.7	0.30	5 (7.0)	0.27

*Alcohol consumption*					
Não	610 (66.1)	70.9±7.9		61 (10.0)	
Sim	313 (33.9)	72.4±8.1	0.01^a^	38 (12.1)	0.32

*Soft drink consumption*					
Não	143 (15.9)	72.2±8.7		21 (14.7)	
Sim	754 (84.1)	71.3±7.9	0.45	78 (10.3)	0.12

*Sleep (sleep well)*					
Não	559 (61.4)	71.3±8.0		58 (10.4)	
Sim	351 (38.6)	71.7±7.9	0.62	39 (11.1)	0.72

*TV time*					
<2h	192 (21.4)	71.6±7.3		15 (7.8)	
≥2h	705 (78.6)	71.4±8.3	0.23	84 (11.9)	0.08

*Computer time*					
<2h	281 (31.3)	71.6±8.7		36 (12.8)	
≥2h	616 (68.7)	71.4±7.8	0.83	63 (10.2)	0.29

*Video game time*					
<2h	646 (72.0)	71±7.9		72 (11.1)	
≥2h	251 (28.0)	72.6±8.3	0.01^a^	27 (10.8)	0.85

*Total screen time*					
<4h	115 (12.8)	70.7±7.9		13 (10.3)	
≥4h	782 (87.2)	71.6±8.1	0.41	86 (11.0)	0.86

*Travel to school*					
Active	448 (49.9)	71.2±7.8		43 (9.6)	
Passive	449 (50.1)	71.7±8.3	0.58	56 (12.5)	0.17

M, mean; SD, standard deviation.

a *p*≤0.05; Mann-Whitney *U* test for independent samples.

Adolescents whose mothers had low education were less likely to have AO in the crude analysis (OR=0.59, 95%CI 0.35-0.98). In the adjusted analysis, the association with maternal education remained (OR=0.56, 95%CI 0.35-0.91), and students who watched television for two hours or more were more likely to have AO (OR=2.11; 95%CI 1.08-4.13) (Table 4).

**Table 4:** *Odds Ratio* and 95% confidence interval, in crude and adjusted binary logistic regression analysis, between abdominal obesity and independent variables.

	Crude analysis			Adjusted analysis^a^	
	OR (CI)	*p*-value		OR (CI)	*p*-value
*Sex* ^b^		0.70			0.71
Male	1			1	
Female	0.90 (0.53-1.53)			1.08 (0.70-1.65)	

*Skin color* ^b^		0.10			0.28
White	1			1	
Black/brown/yellow/red	1.50 (0.92-2.45)			1.26 (0.82-1.93)	

*Age (years)* ^b^		0.44			0.49
14-16	0.82 (0.50-1.35)			0.86 (0.56-1.31)	
17-19	1			1	

*Maternal education* ^c^		0.04^d^			0.02^d^
<8 years	0.59 (0.35-0.98)			0.56 (0.35-0.91)	
≥8 years	1			1	

*Economic level* ^c^		0.06			0.07
High	1			1	
Low	1.65 (0.97-2.78)			0.64 (0.39-1.06)	

*Shift* ^c^		0.45			0.49
Day	1			1	
Evening	1.22 (0.72-2.07)			1.18 (0.72-1.93)	

*Physical activity* ^e^		0.66			0.71
Insufficiently active	1.24 (0.46-3.35)			1.19 (0.45-3.15)	
Active	1			1	

*Alcohol consumption* ^e^		0.56			0.37
No	1			1	
Yes	1.16 (0.69-1.93)			1.24 (0.76-2.03)	

*Soft drink consumption* ^e^		0.08			0.11
No	1			1	
Yes	0.57 (0.30-1.07)			0.62 (0.34-1.12)	

*Sleep (sleep well)* ^e^		0.34			0.46
No	0.78 (0.47-1.29)			0.83 (0.51-1.35)	
Yes	1			1	

*TV time* ^e^		0.10			0.02^d^
<2h	1			1	
≥2h	1.82 (0.88-3.73)			2.11 (1.08-4.13)	

*Computer time* ^e^		0.09			0.09
<2h	1			1	
≥2h	0.61 (0.35-1.08)			0.66 (0.40-1.07)	

*Video game time* ^e^		0.57			0.78
<2h	1			1	
≥2h	0.84 (0.46-1.52)			0.92 (0.53-1.60)	

*Total screen time* ^e^		0.44			0.54
<4h	1			1	
≥4h	1.40 (0.59-3.36)			1.29 (0.55-3.01)	

*Travel to school* ^e^		0.06			0.09
Active	1			1	
Passive	1.60 (0.97-2.65)			1.51 (0.93-2.44)	

OR, odds ratio; CI, confidence interval.

a Adjusted analysis for all independent variables.

b Distal level to outcome variables.

c Intermediate level variables.

d *p*≤0.05.

e Proximal level to outcome variables.

## Discussion

The present study main findings were: (i) about one in 10 adolescents had AO (10.6%); (ii) adolescents who watched television for two or more hours were more likely to have AO (OR=2.11, 95%CI 1.08-4.13), and (iii) adolescents whose mothers had less than eight years of education were less likely to have AO (OR=0.56; 95%CI 0.35-0.91). Previous studies in the same area described AO prevalence of 6.3%,^7^ 13.3%,^9^ and 12.8%.^8^ This study brings as advance the information that sedentary behavior was the main lifestyle factor associated with AO. Such behavior is modifiable through health education initiatives, which may result in lower prevalence of AO. In addition to this variable, the only sociodemographic factor associated with AO was high maternal education, which shows the need for specific investigations for this indicator.

The 10.6% prevalence of AO found in this study is below the values found for adolescents in Spain (11.6%)^5^ and USA (34.7%),^1^ and similar to those found for South Korean adolescents (8.4%).^1^ When compared to studies performed in Brazil, the found prevalence of AO was higher than the values reported for Minas Gerais (Januária) (2.1%)^7^ and lower than the values reported for Maranhão (22.7%)^6^ and Curitiba (12.2%).^8^ These differences in OA prevalence may demonstrate cultural and social differences and similarities,^4^ if we take into account that São José and Curitiba have the human development index ranked as very high (0.809 and 0.823)^16^ and Januária ranked as medium (0.658).^16^

Another factor that may explain the discrepancy between the prevalence found is the use of different cutoff points by different studies. Similar to the present investigation, three studies^5^^-^ 7 used the cutoff points proposed by Taylor et al.^3^ , which defined AO values with Z score ≥1. The validation of WC measurement was performed using dual energy X-ray absorptiometry (DXA) and showed high correlation for both genders (*r*=0.92), with 84% sensitivity and 94% specificity for girls and 87% sensitivity and 92%f specificity for boys.^3^ Unlike these studies, three other studies^1^^,^ 8 ^,^ 9 used different WC percentiles. AO diagnosis depends on the cutoff points used with high sensitivity and specificity values for the population studied. The use of references that do not adopt these criteria may lead to misclassification and underestimate or overestimate the values.^4^

Adolescents whose mothers had less than eight years of education were less likely to have AO. Parental education determines the children's chance of education and family cultural sphere.^25^ Due to the positive relationship with the family income, the higher the educational level, the higher the household income.^25^ Thus, this finding is in line with studies reporting association between AO and high economic level, which is related to obesogenic environments.^4^^,^ 6 ^,^ 7 ^,^ 11 In our study, when the economic level of adolescents was directly investigated, there was no association with the prevalence of AO. This fact shows the importance of assessing factors associated with AO in different ways to determine the presence obesogenic environments.

Television time equal to or greater than 2h was associated with AO. The result of this study is similar to that found by Byun et al.^26^ who reported the association of AO and SB. This association may be explained by the lower energy expenditure throughout the day in adolescents who have greater involvement in SB.^27^ Moreover, the literature has shown that high-calorie food consumption occurs concomitantly with the act of watching television.^27^

WC was higher in male compared to female adolescents, but these differences were not sustained when assessing the prevalence of AO. Moraes et al.^4^ found that, although there is a tendency to higher WC values in men, the association between AO and sex is not yet clear in adolescents. Higher values of WC in male adolescents occur as a result of sexual dimorphism in fat distribution.^10^ Female adolescents, even with a higher percentage of body fat due to hormonal differences between the sexes, have increased accumulation of adipose tissue in the hip and decreased in the waist area compared with their male peers.^10^

Adolescents who played video games for 2h or more had higher WC, which can be explained by the higher WC in male adolescent, as they have video game screen time higher than female.^26^^,^ 28 In our study, boys had higher average of video game screen time (171.5±257.6min) than girls (53.9±157.7min). Moreover, the percentage of adolescents who played video games for 2h or more was higher in males (45.5%) than females (15.5%) (Data not shown in table/figures).

Older adolescents had higher WC values. The literature states that, due to the morphological and physiological development, the WC increases with age and stage of sexual maturation, regardless of the presence of AO.^3^^,^ 10 Another finding of our study was that adolescents with higher alcohol consumption also had higher WC. Epidemiological analysis indicated that alcohol consumption increases with age.^29^ As older students had higher WC values, age can be a confounding variable in the association between alcohol consumption and WC.

Regarding school shift, higher WC was found in adolescents attending evening classes. It is speculated that this finding is related to the fact that these adolescents are older, have low economic levels and, therefore, they work. Thus, they have lifestyle habits more similar to adults and greater engagement in SB.^22^

The study with adolescents attending public high school in São José was a limitation, as it implies that the results may not be extrapolated to private school students who, in Brazil, have different socioeconomic characteristics from those observed in young people from public schools. The strengths of this study were the school-based sample and the use of cutoff points validated through a method with strong correlation with benchmarks in abdominal fat evaluation.^3^ Additionally, several Brazilian studies used the same cutoff points,^6^^,^ 7 which facilitates comparison between studies.

It can be concluded that a high prevalence of abdominal obesity was found in approximately one out of 10 adolescents. Maternal education (≥8 years) and the time sitting in front of television (≥2h) were associated with abdominal obesity.

## Funding

This study did not receive funding.
